# Inhibition of Cardiac RIP3 Mitigates Early Reperfusion Injury and Calcium-Induced Mitochondrial Swelling without Altering Necroptotic Signalling

**DOI:** 10.3390/ijms22157983

**Published:** 2021-07-26

**Authors:** Csaba Horvath, Megan Young, Izabela Jarabicova, Lucia Kindernay, Kristina Ferenczyova, Tanya Ravingerova, Martin Lewis, M. Saadeh Suleiman, Adriana Adameova

**Affiliations:** 1 Department of Pharmacology and Toxicology, Faculty of Pharmacy, Comenius University in Bratislava, 81499 Bratislava, Slovakia; csaba125@gmail.com (C.H.); izabela.jarabicova@gmail.com (I.J.); 2 Faculty of Health Sciences, Bristol Heart Institute, The Bristol Medical School, University of Bristol, Bristol BS8 1TH, UK; my13329@bristol.ac.uk (M.Y.); martin.lewis@bristol.ac.uk (M.L.); M.S.Suleiman@bristol.ac.uk (M.S.S.); 3 Centre of Experimental Medicine, Institute for Heart Research, Slovak Academy of Sciences, 81438 Bratislava, Slovakia; lucia.griecsova@gmail.com (L.K.); kristina.ferenczyova@gmail.com (K.F.); usrdravi@savba.sk (T.R.)

**Keywords:** receptor-interacting protein kinase 3, necroptosis, myocardial ischemia/reperfusion

## Abstract

Receptor-interacting protein kinase 3 (RIP3) is a convergence point of multiple signalling pathways, including necroptosis, inflammation and oxidative stress; however, it is completely unknown whether it underlies acute myocardial ischemia/reperfusion (I/R) injury. Langendorff-perfused rat hearts subjected to 30 min ischemia followed by 10 min reperfusion exhibited compromised cardiac function which was not abrogated by pharmacological intervention of RIP3 inhibition. An immunoblotting analysis revealed that the detrimental effects of I/R were unlikely mediated by necroptotic cell death, since neither the canonical RIP3–MLKL pathway (mixed lineage kinase-like pseudokinase) nor the proposed non-canonical molecular axes involving CaMKIIδ–mPTP (calcium/calmodulin-dependent protein kinase IIδ–mitochondrial permeability transition pore), PGAM5–Drp1 (phosphoglycerate mutase 5–dynamin-related protein 1) and JNK–BNIP3 (c-Jun N-terminal kinase–BCL2-interacting protein 3) were activated. Similarly, we found no evidence of the involvement of NLRP3 inflammasome signalling (NOD-, LRR- and pyrin domain-containing protein 3) in such injury. RIP3 inhibition prevented the plasma membrane rupture and delayed mPTP opening which was associated with the modulation of xanthin oxidase (XO) and manganese superoxide dismutase (MnSOD). Taken together, this is the first study indicating that RIP3 regulates early reperfusion injury via oxidative stress- and mitochondrial activity-related effects, rather than cell loss due to necroptosis.

## 1. Introduction

Myocardial ischemia/reperfusion (I/R) injury is a complex of cellular and molecular pathomechanisms leading to the loss of viable cardiomyocytes and compromise cardiac function [1,2]. Traditionally, necrosis, apoptosis and autophagy have been considered as the main cell death modes underlying cardiac damage under conditions of I/R [3,4]. In recent years, this paradigm has changed and other necrosis-like cell death modes (e.g., necroptosis) have been identified. Necroptosis, a cell death mode linking the RIP1 (receptor-interacting protein kinase 1)–RIP3–MLKL (mixed lineage kinase domain-like pseudokinase) axis [5,6], has been reported in several models of acute myocardial I/R injury [7,8,9,10,11]. Several studies have challenged the involvement of RIP1 in necroptosis signalling [12,13], thereby indicating a major role for the RIP3–MLKL axis. These proteins are activated and, thus, advance necroptosis execution by phosphorylation modifications. RIP3 can be activated by RIP1 [6] or is autoactivated upon dimerization and subsequent autophosphorylation at Ser229/Thr232 [14] while MLKL is activated by RIP3 at Thr357 and Ser358 residues [15]. As a result, they cause cytoplasmic membrane rupture [16] due to alterations in ion homeostasis [17], the activation of proteases of the ADAMs (disintegrins and metalloproteinases) family [18] and oxidative stress [19]. In addition to such deleterious effects of RIP3–MLKL heterooligomers and MLKL homooligomers within the plasma membrane, there are also indications suggesting that these protein complexes can interact with certain mitochondrial molecules, thereby connecting the necroptotic process to the mitochondria [20]. Indeed, non-canonical signalling pathways, such as the RIP3-mediated activation of CaMKIIδ (Ca2+/calmodulin-dependent protein kinase II delta) promoting mPTP (mitochondrial permeability transition pore) opening [21] and/or RIP3–PGAM5 (phosphoglycerate mutase family member 5)–Drp1 (dynamin-related protein 1) mediated mitochondrial fission [22], have been reported. In line, CaMKII inhibition [23,24] as well as PGAM5 inhibition [22] have been shown to elicit cardioprotective effects in acute models of myocardial I/R by mitigating necrotic and necroptotic cell death. A deficiency of RIP3—an upstream signal molecule of CaMKIIδ and PGAM5—has been associated with a significantly better ejection fraction and less post-ischemic adverse remodelling after chronic left anterior descending coronary artery ligation [8]. However, it is completely unknown whether a pharmacological approach of an inhibition of RIP3 is also capable of alleviating heart injury due to acute I/R and whether it is due to targeting the non-canonical mitochondria-associated pathways. Likewise, because RIP3 has also been linked with inflammation and ROS (reactive oxygen species) generation [8,21,25,26] and thereby acting as a convergent point of multiple pathways promoting heart damage, it is possible that such intervention can also be targeting other intracellular signalling pathways.

Thus, in view of the lack of information regarding the status of RIP3 in acute myocardial I/R injury, we treated rat hearts with an RIP3 inhibitor. We hypothesized that the inhibition of RIP3, positioned upstream of PGAM5–Drp1 and/or CaMKIIδ–mPTP axes, would dampen the heart damage even in the early phase of reperfusion, which is the most critical period for injury and mitochondrial swelling due to an excessive ROS production [27]. Other possible underlying molecular signalling pathways were also examined.

## 2. Results

### 2.1. LDH Release

**Figure 1:**
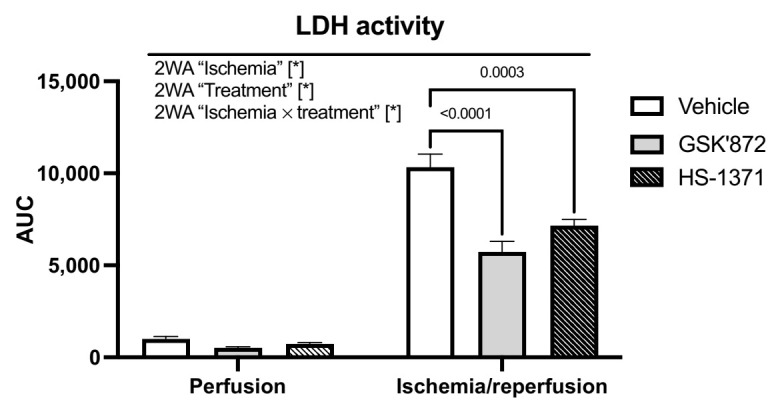
Analysis of the effect of RIP3 inhibition on LDH activity. Data are presented as mean ± SEM; * *p* < 0.05. 2WA—two-way ANOVA; “Ischemia”—presence of ischemia/reperfusion; “Treatment”—presence of GSK’872 or HS-1371; “Ischemia × treatment”—interaction of the two factors.

Myocardial I/R injury was associated with an increased LDH release and the inhibition of RIP3 (GSK’872 or HS-1371) significantly mitigated LDH release, thereby indicating RIP3 involvement in triggering necrosis-like cell death (Figure 1).

### 2.2. mPTP Opening

**Figure 2:**
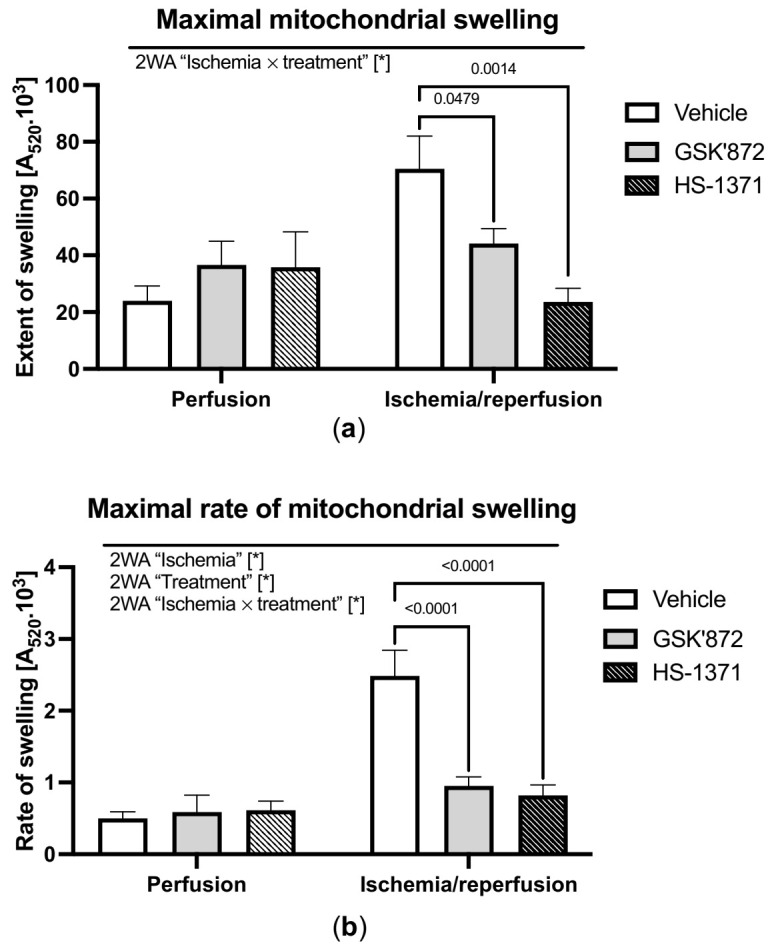
Analysis of the effect of RIP3 inhibition on mitochondrial swelling. (**a**) Maximal mitochondrial swelling evaluated as the maximum decrease in A520 after calcium addition to de-energised mitochondria; (**b**) maximal rate of mitochondrial swelling evaluated as the rate of decrease in A520 in the first 5 s after calcium addition to de-energised mitochondria. Data are presented as mean ± SEM; * *p* < 0.05. 2WA—two-way ANOVA; “Ischemia”—presence of ischemia/reperfusion; “Treatment”—presence of GSK’872 or HS-1371; “Ischemia × treatment”—interaction of the two factors.

In the absence of ischemic insult, the maximal extent of mitochondrial swelling was unaffected by either of the used RIP3 inhibitors. On the other hand, during early reperfusion, both drugs mitigated the I/R-mediated increase in calcium-induced mPTP opening (Figure 2). A similar pattern of results was observed in the maximal rate of mitochondrial swelling—another important parameter of the mPTP activity [28,29] (Figure 2). Thus, these data indicated a conspicuous RIP3-dependent regulation of calcium-sensitivity of the mPTP.

### 2.3. Hemodynamic Parameters of the Heart and Arrhythmia Triggering

As both RIP3 inhibitors comparably affected LDH release, mPTP opening and because GSK’872 has an advanced pharmacologic profile in comparison to HS-1371, only the former inhibitor was used in further studies focused on the assessment of cardiac function and molecular analysis.

**Figure 3:**
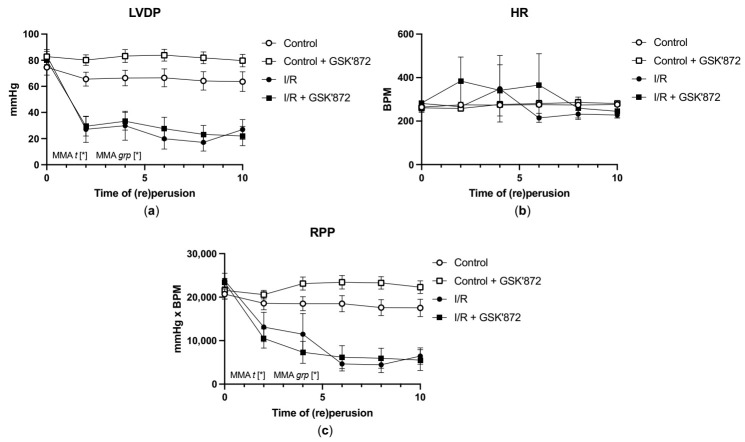
Time-course of the selected hemodynamic parameters of the heart. (**a**) LVDP, (**b**) HR, (**c**) RPP. Data are presented as mean ± SEM; * *p* < 0.05. MMA—mixed-model ANOVA; *t*—time; *grp*—group.

The baseline hemodynamic data and hemodynamic data after 10 min of reperfusion are shown in Table 1. As expected, adverse effects of ischemia were evidenced by a significantly decreased LVDP from 83.4 ± 4.7 at baseline to 26.9 ± 6.4 mmHg at the end of reperfusion (*p* < 0.05), +(dP/dt)max from 1749.5 ± 106.6 to 376.6 ± 111.0 mmHg/s, and −(dP/dt)max from 1397.4 ± 66.6 to 342.9 ± 111.1 mmHg/s (*p* < 0.05), respectively. This was accompanied by a simultaneous increase in LVEDP from 3.53 ± 1.0 mmHg at baseline to 31.7 ± 7.8 mmHg at the end of reperfusion (*p* < 0.05), (Table 1). GSK’872 failed to ameliorate such detrimental effects during the whole reperfusion period (Table 1, Figure 3). HR was unaffected by the pharmacological intervention. Thus, RPP (rate pressure product), considered as the main parameter characterizing haemodynamic function, was not altered by RIP3 inhibition during the entire experiment either (Figure 3). In line, the arrhythmic score after I/R injury was comparable between the treated and non-treated group (data not shown).

### 2.4. Molecular Analyses of Canonical and Non-Canonical Signalling Pathways of Necroptosis

**Figure 4:**
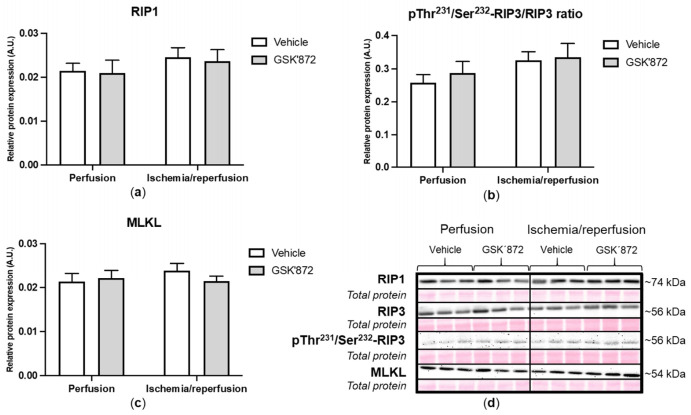
Analysis of activation of canonical necroptotic pathway in left ventricles lysates. Immunoblot quantification of RIP1 (**a**), pThr^231^/Ser^232^-RIP3/RIP3 ratio (**b**) and MLKL (**c**); representative immunoblots and total protein staining (**d**). Data are presented as mean ± SEM.

**Figure 5:**
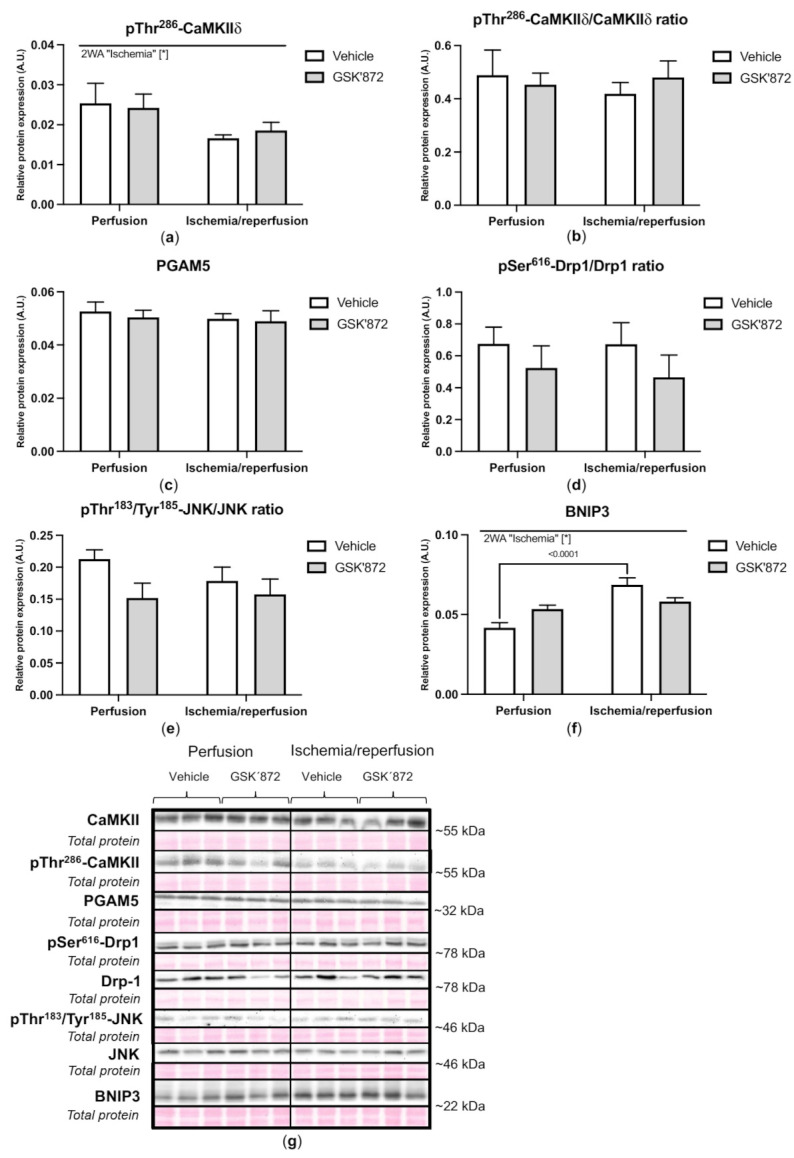
Analysis of activation of non-conventional necroptotic pathways in left ventricles lysates. Immunoblot quantification of pThr^286^-CaMKIIδ (**a**), pThr^286^-CaMKIIδ/CaMKIIδ ratio (**b**), PGAM5 (**c**), pSer^616^-Drp1/Drp1 ratio (**d**), pThr^183^/Tyr^185^-JNK/JNK ratio (**e**) and BNIP3 (**f**); representative immunoblots and total protein staining (**g**). Data are presented as mean ± SEM; * *p* < 0.05. 2WA—two-way ANOVA; “Ischemia”—presence of ischemia/reperfusion.

Molecular analyses of the investigated necroptotic signalling pathways are indicated in Figure 4 and Figure 5. In conditions of 30 min ischemia followed by 10 min reperfusion, the canonical necroptotic signalling pathway did not seem to be activated. In fact, the expression of none of the RIP kinase family (RIP1 and RIP3), the pro-necroptotic phosphorylation of RIP3 on Thr231/Ser232, nor the main executive necroptotic protein MLKL were changed due to the applied I/R protocol or RIP3 inhibition (Figure 4). Likewise, we did not find any changes in the expression of caspase-8 which supresses RIP1–RIP3 kinase complex-dependent necroptosis and is critical for the death receptor-induced apoptosis. In line, the levels of main markers of apoptosis did not differ among groups being subjected either to I/R or treatment (Figure S1).

The activation of the non-canonical pathways being associated with RIP3 and mitochondria was not clearly proved under settings of I/R either. Paradoxically, pThr286-CaMKIIδ levels, which can interfere with mPTP opening [21,30], were downregulated by I/R and this effect was not reversed by GSK’872 (Figure 5). Furthermore, an immunoblotting analysis of both PGAM5 and its mitochondrial fission-mediating target protein Drp1 [31], revealed no changes in their expression due to either I/R injury or treatment. A non-significant decreased phosphorylation of Drp1 on Ser616 was observed in the GSK’872-treated groups regardless of ischemic insult (Figure 5).

Neither I/R- nor GSK’872-mediated changes in the total JNK levels (c-Jun N-terminal kinase) were observed. However, a slight decrease in pThr183/Tyr185-JNK levels was seen due to GSK’872 treatment in both ischemic, and non-ischemic hearts. The expression of BNIP3 (Bcl2 interacting protein 3) was significantly increased by I/R. GSK’872 tended to decrease these levels (Figure 5).

Collectively, these molecular data did not indicate the activation of necroptotic cell death under settings of 30 min ischemia followed by 10 min reperfusion and RIP3 inhibition modified none of the investigated necroptotic signalling pathways. Thus, the protective effects of GSK’872 on myocardial I/R injury are likely mediated by the interference with other pathomechanisms, such as oxidative stress, which significantly contributes to mitochondrial swelling [27,32] as well as inflammation, which accompanies necrosis-like cell death modes [25,33]. In addition, because these two mechanisms have also been linked to RIP3 [21,25], we examined the expression of some ROS-producing enzymes and markers of inflammatory response.

**Figure 6:**
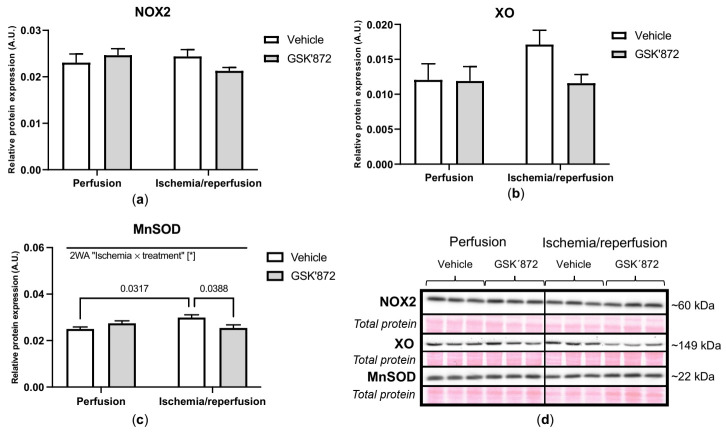
Analysis of oxidative stress in left ventricles lysates; immunoblot quantification of NOX2 (**a**), XO (**b**), MnSOD (**c**); representative immunoblots and total protein staining (**d**). Data are presented as mean ± SEM; * *p* < 0.05. 2WA—two-way ANOVA; “Ischemia × treatment”—interaction of the presence of ischemia/reperfusion and GSK’872.

Neither the investigated I/R protocol, nor treatment caused a significant change in the expression of the pro-oxidative enzymes NOX2 (NADPH oxidase 2) and XO (xanthine oxidase). On the contrary, I/R elevated the levels of MnSOD (manganese superoxide dismutase) and such an increase was reversed by GSK’872 (Figure 6).

**Figure 7:**
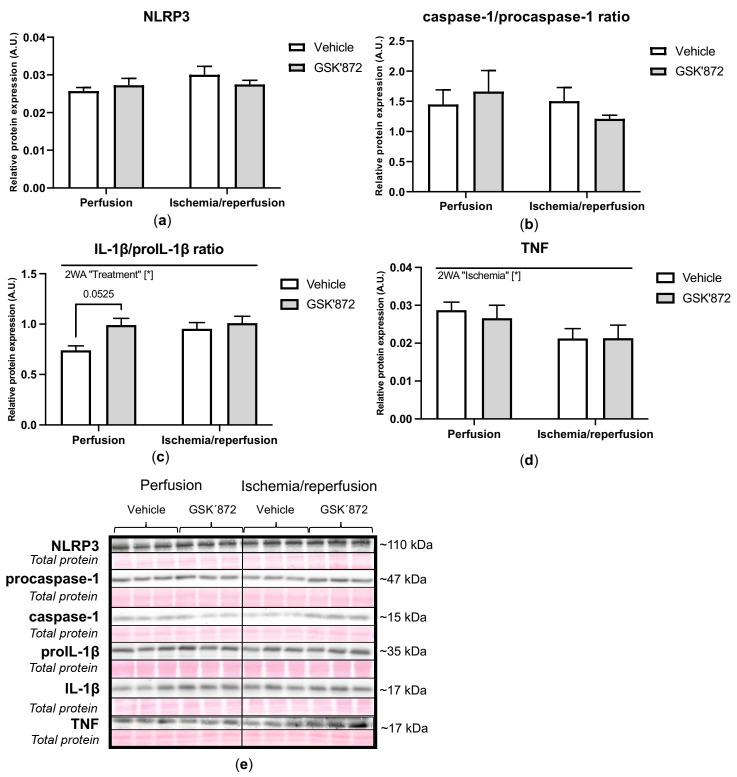
Analysis of inflammation in left ventricles lysates; immunoblot quantification of NLRP3 (**a**), caspase-1/procaspase-1 ratio (**b**), IL-1 β/proIL-1β ratio (**c**), TNF (**d**); representative immunoblots and total protein staining (**e**). Data are presented as mean ± SEM; * *p* < 0.05. 2WA—two-way ANOVA; “Ischemia”—presence of ischemia/reperfusion; “Treatment”—presence of GSK’872.

The expression of pro-inflammatory markers such as NLRP3 (NOD-, LRR- and pyrin domain-containing protein 3) and caspase-1 was comparable between the experimental groups. Similarly, ischemia did not induce any changes in the cleavage of IL-1β (interleukin-1 beta); however, GSK’872 elevated such cleavage under non-ischemic conditions reaching borderline statistical significance (*p* = 0.0525). Intriguingly, I/R caused a decrease in the TNF (tumour necrosis factor) level, which was not altered by GSK’872 (Figure 7).

## 3. Discussion

The present study was carried out to extend the knowledge about necroptotic signalling in I/R hearts and examine a crucial role of RIP3 as the convergence point of potential multiple signalling pathways producing such injury. We showed for the first time that despite the observed plasma membrane rupture of cardiac cells evidenced by a higher LDH release, the early reperfusion phase (10 min) is not likely to involve the activation and execution of necroptosis via the canonical RIP3–MLKL molecular pathway. Other RIP3-associated necroptotic pathways such as the RIP3-mediated activation of CaMKIIδ [21] and the RIP3–PGAM5–Drp1 axis [22] did not seem to be activated due to the applied I/R protocol either. Similarly, I/R did not influence the phosphorylation of JNK on Thr183/Tyr185, but conversely caused an increase in BNIP3 expression, a stimulator of mitochondria-mediated cell death [34,35]. GSK’872, an RIP3 inhibitor, markedly mitigated the rupture of the plasma membrane and prevented mPTP opening. However, these beneficial effects were not accompanied by any significant impact on the investigated signalling pathways of necroptosis execution and did not result in the abrogation of I/R-induced cardiac dysfunction. Other than GSK’872, comparable effects on the calcium-sensitive opening of mPTP, a marker of mitochondria swelling, and on LDH release, were also observed after treatment with another RIP3 inhibitor—HS-1371. Therefore, this study convincingly indicates the involvement of RIP3 in myocardial injury due to short reperfusion following ischemia, while the underlying mechanism likely originates from non-necroptotic action of this protein kinase.

In our previous study investigating a more prolonged reperfusion period (40 min), the execution of necroptosis via its canonical pathway was evident and was associated with post-ischemic systolic and diastolic dysfunction [11]. Likewise, other authors have reported necroptosis under settings of a longer reperfusion period (120 min till 4 h) of a previously ischemic heart as well as in post-ischemic cardiomyopathy [10,36,37]. In contrast, in this study, the upregulation of neither markers of necroptosis was detected, thereby supporting the theory that the MLKL-driven execution of necroptosis might occur in later phases of reperfusion. Results from this study also suggest that the mechanical function of the heart is unlikely significantly affected by necroptotic cell death. In fact, changes in LVDP and LVEDP due to I/R indicated cardiac dysfunction despite the absence of necroptosis activation. The same is true for RIP3 inhibitor-treated I/R hearts, although such pharmacologic intervention mitigated the lysis of cardiac cells. The failure of protective effects of RIP3 inhibition in I/R injury with respect to hemodynamic parameters of the heart can be explained by the fact that necroptosis has not been fully executed. No modulation of plausible off-target proteins of RIP3 being associated with the regulation of excitation-contraction coupling, however, can also provide a certain explanation. In fact, some necroptosis inhibitors targeting RIP1 have shown the ability to mitigate myocardial I/R injury due to the retardation of necroptosis [9,11,37] and to affect the phosphorylation of pThr286-CaMKIIδ, pThr17-PLN (phospholamban) and pSer282-cMyBPc (cardiac myosin-binding protein c), as well as nitrosative stress [38].

Although RIP3 inhibition has been shown to be protective in a necroptotic death- and inflammation-limiting manner in various in vivo and in vitro models of cerebral [39,40] and hepatic ischemia [41], to the best of our knowledge, the direct effects of this inhibitor on myocardial function after I/R have not been studied yet. On the other hand, the genetic approach targeting RIP3—RIP3 depletion led to the mitigation of inflammation four days after myocardial infarction as evidenced by a significantly decreased invasion of CD3-positive cells [8]. Therefore, under such conditions of ischemia followed by 4-day reperfusion, RIP3 seems to be detrimental in a dichotomous MLKL-driven, pro-necroptotic and pro-inflammatory mode. Likewise, RIP3 activation has been suggested to underlie tissue structural and histological abnormalities induced in post-infarction heart failure due to the promotion of both inflammation and necroptosis [42]. Conversely, RIP3 likely mediates early tissue damage via other mechanisms, independently of the canonical or the other proposed necroptotic signalling pathways as indicated in this study. In support, it has been reported that infarct size is not altered in RIP3-/- hearts 24 h after the left anterior descending artery ligation in comparison with control hearts [8]. Thus, in such hearts lacking RIP3, a significantly improved ejection fraction with the simultaneous prevention of hypertrophy reported 30 days after myocardial infarction [8], may be mediated by the complex action of RIP3 involving the interference with inflammation, oxidative stress and mitochondria along with the regulation of necroptosis. This theory about the action of RIP3 in short-term and long-term I/R settings could also provide another explanation for the lack of cardioprotection of RIP3 inhibition with respect to the hemodynamic data shown in this study. In fact, the applied I/R protocol with a short reperfusion phase did not lead to inflammatory response, as evidenced by no alterations in NLRP3, caspase-1 and IL-1β. Interestingly, TNF was decreased under ischemic conditions. The pharmacologic intervention used in our study influenced neither of these inflammatory markers except for a borderline significant GSK’872-mediated increase in IL-1β cleavage under non-ischemic conditions. Therefore, no clear evidence of RIP3-mediated action on inflammation during such acute I/R settings was reported in this study.

Mitochondria are essential regulators of cellular energy metabolism; however, under pathophysiologic conditions they are posited as critical multifaceted executioners of cell death [43,44]. Under I/R conditions, mitochondria particularly contribute to necrotic death by either a higher uptake/decreased release of Ca^2+^ to/from the mitochondrial matrix or increased levels of oxidants [45,46]. One of the key processes of how cells prevent such exuberant mitochondrial ROS generation is by initiating mitophagy—a specific form of autophagy responsible for selective clearance of damaged mitochondria to prevent further cellular oxidative damage [47]. The pioneer study investigating the possible involvement of RIP3 in the process of mitophagy after cardiac I/R injury has indicated that this protein kinase limited the extent of mitophagy by suppressing FUNDC1 (FUN14 Domain Containing 1)—an outer mitochondrial membrane protein acting as a mitophagy receptor—and this in turn triggered apoptotic loss of cardiomyocytes [48]. More recently, another group of authors confirmed RIP3-mediated limitation of mitophagy, however, via a different mechanism [49]. Zhu et al. have shown that RIP3 suppressed the AMPK (5’ adenosine monophosphate-activated protein kinase)/Parkin-mediated mitophagy which consequently resulted in massive mPTP opening and the initiation of necroptosis [49]. In view of these findings, such a protective effect of RIP3 inhibition on the maximal extent and rate of mPTP opening observed in our study might be mediated, at least in part, by the disruption of the RIP3-mediated suppression of mitophagy. This hypothesis along with describing the precise mechanism of mitochondria interaction with necroptosis, however, warrants further research. On the other hand, early studies investigating the relevance of necroptosis during I/R injury pointed out that the cardioprotective effect of necroptosis inhibition by necrostatin-1, a RIP1 inhibitor, is accompanied by a delayed mPTP opening in a cyclophilin D-dependent manner [50,51]. However, the later discovered fact that RIP1 is dispensable for necroptosis and RIP3 alone is sufficient to activate the execution of this form of programmed necrosis has advanced the view on necroptosis signalling and highlighted a central role of the latter kinase [14]. Such recognition even led to some clinical translation, since it has been shown that the plasma RIP3 levels correlate with the severity of coronary artery disease, reaching the peak after acute myocardial infarction [52]. A similar correlation was found in the case of heart failure progression [53]. Therefore, focusing on targeting RIP3 kinase might have greater potential from the preventive and therapeutic point of view. In the present study, we clearly showed that RIP3 is involved in mitochondrial swelling via the effect on mPTP opening under acute reperfusion injury. Since we investigated de-energised mitochondria with buffered (Ca^2+^) accompanied by calcium-ionophore (A23187), the GSK’872-mediated decrease in mPTP opening must reflect a change in calcium-sensitivity of the mPTP and cannot be caused by differences in basal mitochondrial calcium loading. The same effect was observed also in case of another RIP3 inhibitor—HS-1371—, which prevents ATP binding while GSK’872 inhibits the kinase domain [54]. The underlying mechanisms of such effects of these RIP3 inhibitors can be explained by either the regulation of the phosphorylation of some mPTP component(s) or the oxidative modification of critical thiol groups on the mPTP, resulting in (Ca^2+^)-sensitisation [55]. Regarding the data about CaMKIIδ phosphorylation, it is unlikely that such a delayed mPTP opening due to RIP3 inhibition is mediated by the regulation of this protein kinase. Thus, ROS-sensitive mPTP opening could be instead responsible for such findings due to RIP3 inhibition. Recently, it has been illustrated that, in the setting of myocardial I/R injury, the RIP3-mediated elevation of endoplasmic reticulum stress resulted in an increased mPTP opening via the XO-mediated ROS production [56]. In our hands, there was an unsignificant elevation of XO due to I/R, while RIP3 inhibition normalized these levels to those seen in non-ischemic, vehicle-perfused hearts. In line, we found out that the I/R-induced increased expression of MnSOD, an enzyme catalysing the dismutation of superoxide anion radical to hydrogen peroxide and molecular oxygen, was normalized by RIP3 inhibitor, supporting the evidence of RIP3 connection with oxidative stress. On the other hand, NOX2 levels were affected due to neither I/R nor RIP3 inhibition. As this pro-oxidant enzyme producing superoxide free radical has also been linked with necroptosis [21], and because necroptosis activation/execution presented in this study were not evident, these findings support the theory that necroptotic injury likely proceeds in later rather than earlier phases of reperfusion. Although we delineated some mechanisms of RIP3-mediated regulation of mPTP opening, other underlying mechanisms cannot be ruled out either. Indeed, mPTP-necrosis, which is suggested to be the most prevalent form of necrosis/necrotic-like loss of cardiac cells after ischemic insult [45], could also be under control of RIP3 inhibition, and, thus, responsible for findings reported in this study.

## 4. Materials and Methods

### 4.1. Chemicals and Reagents

Drugs used (GSK2399872A; HS-1371) were sourced from Selleck Chemicals (Pittsburgh, PA, USA). Unless otherwise specified, all other chemicals used were supplied by CENTRALCHEM (Bratislava, Slovakia), Merck (Darmstadt, Germany), Apollo Scientific (Stockport, UK), Sigma-Aldrich (Darmstadt, Germany) or Alfa Aesar (Haverhill, MA, USA) in the highest available purity.

### 4.2. Animals and Experimental Groups

**Figure 8:**
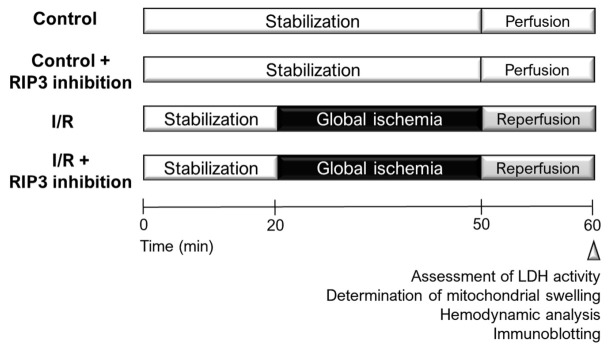
An illustration showing experimental design. RIP3 inhibitors—GSK’872 or HS-1371—were added during the whole 10 min reperfusion.

All housing conditions and experimental protocols were approved by both the Ethics Committee of the Faculty of Pharmacy, Comenius University in Bratislava, Slovak Republic and the University of Bristol Animal Welfare Ethical Review Board (ethical approval number UB/15/017). All procedures described herein were performed in accordance with both the Guide for the care and Use of Laboratory Animals, published by the US National Institutes of Health (Guide, NRC 2011) and approved by the Animal Health and Welfare Division of the State Veterinary and Food Administration of the Slovak Republic and also in a manner compliant with the Animals (Scientific Procedures) Act 1986 of the United Kingdom and consistent with the Guide for the Care and Use of Laboratory Animals (1996, published by the National Academy Press, 2101 Constitution Ave. NW, Washington, DC, USA). Adult male Wistar rats (14–16 weeks old; 250–260 g) were supplied by either Charles River Laboratories (Oxford, UK) for mitochondrial and LDH (lactate dehydrogenase) measurements or the Institute of Experimental Pharmacology & Toxicology, Department of Toxicology and Laboratory Animals Breeding (Dobrá Voda, SR) for hemodynamic and molecular analyses. All animals were housed for 4–7 days at 23 ± 1 °C, with a relative humidity of 60–70% and a light/dark cycle of 12 h, with free access to water and standard rat chow. After adaptation, animals were randomized into one of the following groups: perfusion only (Control); control + GSK’872; control + HS-1371; I/R; I/R + GSK’872; I/R + HS-1371. Control and I/R were treated with a vehicle (0.004% *v*/*v* DMSO). Two structurally different RIP3 inhibitors—GSK’872 (250 nmol/L) or HS-1371 (50 nmol/L)—dissolved in DMSO stock, were used at an equipotent dose and given during a 10 min long perfusion/reperfusion period (Figure 8).

### 4.3. Experimental Myocardial I/R Protocol

Rats were sacrificed by either stunning followed by cervical dislocation for mitochondrial and LDH measurements or by anaesthesia induced with i.p. injection of sodium pentobarbital (60 mg/kg) in combination with heparin for hemodynamic and molecular analysis. Following both procedures, hearts were quickly removed, placed in ice-cold perfusion buffer and retrograde perfused in Langendorff mode. Perfusion with a modified Krebs–Henseleit buffer (95% O_2_, 5% CO_2_, mmol/L: NaCl 118, KCl 4.8, MgSO_4_ 1.2, NaHCO_3_ 25, KH_2_PO_4_ 1.18, CaCl_2_ 1.25, glucose 11; pH 7.4) was performed at conditions of the constant coronary flow rate 10 mL/min for mitochondrial measurements, while hearts for hemodynamic and molecular investigation were perfused at a constant perfusion pressure of 73 mmHg, both at a temperature of 37.5 ± 0.2 °C. After time-matched aerobic perfusion, a 30 min global ischemia was induced by clamping of aortic flow and was followed by a 10 min reperfusion period. Cardiac function evidenced by LVDP (left ventricular developed pressure), LVEDP (LV end-diastolic pressure), maximal rates of pressure development (+(dP/dt)max) and fall (−(dP/dt)max) and HR (heart rate), was evaluated as described previously [38] using PowerLab/8SP Chart 8 software. Ventricular tissue samples of the hearts were rapidly homogenised for mitochondria isolation. LV tissue samples for molecular analysis were excised, immediately frozen in liquid nitrogen and stored at −80 °C until further processing.

### 4.4. Determination of LDH Activity

The effluent perfusate collected from the hearts of all groups during the whole 10 min long reperfusion was used for LDH activity determination. A protocol of Bergmeyer H–U 1963 with some modifications was followed. Briefly, 80 μL of the sample was added to 910 μL of the buffer (pH 7.4) containing 100 mmol/L triethanolamine and 100 μmol/L reduced β-NADH. After addition of 10 μL of 0.1 mol/L sodium pyruvate to the reaction mixture, the change of absorbance at 340 nm (A340) was recorded using a spectrophotometer over 5 min at 37 °C. LDH activity was calculated according to the rate of A340 decrease.

### 4.5. Isolation of Mitochondrial Fractions

All procedures were carried out either on ice or in the cold room (0–4 °C), with pre-chilled instruments, reagents, and centrifuge (4 °C). Following Langendorff perfusion, the heart was removed from the aortic cannula and cut rapidly into small pieces. The tissue was then immersed in ice-cold sucrose buffer (mmol/L: sucrose 300, Tris-HCl 10, EGTA 1; pH 7.2) and homogenized with a Polytron homogenizer at setting 3 for 5 s. The homogenate was rapidly diluted to 30 mL with isolation buffer containing 5 mg/mL bovine serum albumin, centrifuged at 2000× *g* for 120 s to remove cell debris. The supernatant was then centrifuged at 10,000× *g* for 5 min yielding a crude particulate fraction which was re-suspended and diluted to 30 mL with isolation buffer (mmol/L: sucrose 300, Tris-HCl 10; pH 7.2). This suspension underwent further centrifugation at 10,000× *g* for 5 min yielding a pellet referring to the mitochondrial fraction, which was re-suspended in 200 μL isolation buffer (mmol/L: sucrose 300, Tris-HCl 10; pH 7.2) and used for measurement of mPTP opening.

### 4.6. Determination of mPTP Opening

The mPTP opening was determined at 37 °C under de-energized conditions by analysing the decrease in light scattering (monitored as A520) that accompanies mitochondrial swelling. Mitochondria at 0.2 mg protein/mL were incubated for 5 min in swelling buffer (mmol/L: KSCN 150, MOPS 20, Tris 10, nitrilotriacetic acid 2, and supplemented with 2 μmol/L A23187, 0.5 μmol/L rotenone and 0.5 μmol/L antimycin A; pH 7.2). Swelling was initiated by addition of 1 mmol/L CaCl_2_ to give a buffered free [Ca^2+^] of 80 μmol/L [29].

### 4.7. SDS-PAGE and Immunoblotting

Left ventricular tissue samples of the hearts were processed for immunoblotting analysis by SDS-PAGE and Western blotting as described previously [42]. Briefly, post-electrophoresis, proteins were transferred onto PVDF membranes (Immobilon-P, Merck Millipore) and incubated with primary antibodies against RIP1 (#3493, Cell Signaling Technology, Danvers, MA, USA), RIP3 (#15828, Cell Signaling Technology, Danvers, MA, USA), pThr231/Ser232-RIP3 (ab222320, Abcam, Cambridge, UK), MLKL (ab243142, Abcam, Cambridge, UK), CaMKIIδ (ab181052, Abcam, Cambridge, UK), pThr286-CaMKIIδ (ab171095, Abcam, Cambridge, UK), PGAM5 (ab244218, Abcam, Cambridge, UK), Drp1 (#5391, Cell Signaling Technology, Danvers, MA, USA), pSer616-Drp1 (#4494, Cell Signaling Technology, Danvers, MA, USA), JNK (#9252, Cell Signaling Technology, Danvers, MA, USA), pThr183/Tyr185-JNK (#9255, Cell Signaling Technology, Danvers, MA, USA), BNIP3 (#3769, Cell Signaling Technology, Danvers, MA, USA), NOX2 (ab129068, Abcam, Cambridge, UK), XO (ab109235, Abcam, Cambridge, UK), MnSOD (#13141, Cell Signaling Technology, Danvers, MA, USA), NLRP3 (#15101, Cell Signaling Technology, Danvers, MA, USA), caspase-1 (ab179515, Abcam, Cambridge, UK), IL-1β (ab9722, Abcam, Cambridge, UK) and TNF (ab66579, Abcam, Cambridge, UK). Subsequently, membranes were incubated with the following HRP-conjugated secondary antibodies: donkey anti-rabbit IgG (711-035-152, Jackson ImmunoResearch, West Grove, PA, USA), donkey anti-mouse IgG (115-035-174, Jackson ImmunoResearch, West Grove, PA, USA), donkey anti-rat IgG (112-035-175, Jackson ImmunoResearch, West Grove, PA, USA) and mouse anti-rat light chain specific IgG-HRP (112-035-175, Jackson ImmunoResearch, West Grove, PA, USA). Signals were detected using enhanced chemiluminescence (Crescendo Luminata, Merck Millipore, Burlington, MA, USA) and captured by a chemiluminescence imaging system (myECL imager, Thermo Scientific, Waltham, MA, USA). Total protein staining of membranes with Ponceau S assessed by scanning densitometry was used as the loading control in total tissue lysates [57]. Relative expression of protein bands of interest was calculated by normalizing the intensity of a protein band with its whole lane protein staining intensity.

### 4.8. Statistical Analysis

Data are expressed as means ± standard error of means (SEM) for the number of animals in the group. Two-way ANOVA (2WA) and Holm–Sidak’s post hoc tests were applied for comparison of differences in variables with normal distribution between the 6 groups (factor “Ischemia”—presence of ischemia/reperfusion; factor “Treatment”—presence of GSK’872 or HS-1371; factor “Ischemia x treatment”—the interaction of the two factors). Mixed-model ANOVA (MMA) was used to compare time-course hemodynamic data of perfusion-only and I/R groups (factor “*t*”—time; factor “*grp*”—group). Extent of LDH leakage was estimated by computing the area under the curve (AUC) of the graph plotting the LDH activity during the reperfusion period. All analyses were performed with GraphPad Prism 8.00 for Windows (GraphPad Software, San Diego, CA, USA). Differences between groups were considered significant when *p* < 0.05 and the specific *p* values are indicated in Figures.

## 5. Conclusions

Taken together, this study indicated for the first time that necroptosis is unlikely to be responsible for compromised cardiac function during early reperfusion. There is no evidence about the activation of either the canonical (RIP3–MLKL) and/or the proposed non-canonical signalling pathways of necroptosis involving CaMKIIδ-mPTP, PGAM5–Drp1 and JNK–BNIP3 axes. Moreover, the inhibition of RIP3 prevented the rupture of the plasma membrane without the ability to modulate the investigated necroptotic signalling and delayed mPTP opening. These effects do not seem to be associated with its upstream regulator—CaMKIIδ—but may be caused rather by the RIP3-mediated regulation of oxidative stress via XO and MnSOD. The proposed linkage of RIP3 with mitochondria and oxidative stress unlikely determines heart function, because RIP3 inhibition did not affect hemodynamic parameters of I/R hearts. In addition, RIP3 inhibition did not affect the NLRP3-inflammasome signalling which, seems to be activated in later phases of reperfusion rather than in an acute phase as we tested. Data collectively suggest that RIP3 interferes with certain signalling pathways being activated in early reperfusion, but without the capability to underlie the main phenotypes of the injury, such as necroptotic cell death and heart dysfunction. RIP3 might be a prominent target for further research, mainly in relation to mitochondrial pathophysiology and oxidative stress.

## Figures and Tables

**Table 1:** Hemodynamic parameters of isolated rat hearts. Data are presented as ± SEM; * *p* < 0.05. MMA—mixed-model ANOVA.

Stabilization							10 min (re) Perfusion					
	LVDP (mmHg)	LVEDP (mmHg)	HR (BPM)	+dP/dt (mmHg/s)	−dP/dt (mmHg/s)	RPP (mmHg × BPM)	LVDP (mmHg)	LVEDP (mmHg)	HR (BPM)	+dP/dt (mmHg/s)	−dP/dt (mmHg/s)	RPP (mmHg × BPM)
Control	74.6 ± 6.1	5.5 ± 1.1	264.0 ± 19.0	1591.6 ± 121.3	1225.9 ± 76.7	20,724.5 ± 1156.9	63.6 ± 7.6	3.4 ± 1.3	276.1 ± 7.2	1387.6 ± 163.4	1047.7 ± 128.6	17,529.6 ± 1978.9
Control + GSK’872	82.8 ± 3.7	4.0 ± 0.4	261.1 ± 21.9	1683.2 ± 78.3	1296.7 ± 59.5	21,595 ± 2009.8	79.7 ± 4.4	1.3 ± 0.6	281.0 ± 11.3	1655.9 ± 100.0	1343.0 ± 80.5	22,324.2 ± 1449.2
IR	83.4 ± 4.7	3.5 ± 1.0	281.3 ± 3.4	1749.5 ± 106.6	1397.4 ± 66.6	23,873.7 ± 1519.0	26.9 ± 6.4 *	31.7 ± 7.8 *	228.1 ± 12.7 *	376.6 ± 110.9 *	342.9 ± 111.1 *	6469.1 ± 1596.7 *
IR + GSK’872	79.9 ± 3.1	3.2 ± 0.9	283.1 ± 7.2	1742.5 ± 70.0	1355.6 ± 48.5	23,352.9 ± 958.9	22.0 ± 7.4 *	34.6 ± 9.5 *	245.1 ± 30.6	376.8 ± 154.8 *	319.5 ± 129.6 *	5549.5 ± 2391.8 *

## Data Availability

Data are contained within the article or Supplementary Materials.

## Supplementary Materials

The following are available online at https://www.mdpi.com/article/10.3390/ijms22157983/s1, Figure S1: Caspase-8 and some apoptosis markers in rat hearts subjected to RIP3 inhibition. Immunoblot quantification of procaspase-8 (a), caspase-8 (b), procaspase-3 (c), Bcl2/Bax ratio (d); Representative immunoblots and total protein staining (e). Data are presented as mean ± SEM; * *p* < 0.05. 2WA—two-way ANOVA; factor “Ischemia”—presence of ischemia/reperfusion.

## Abbreviations

ADAMs	disintegrins and metalloproteinases
AMPK	5’ adenosine monophosphate-activated protein kinase
BNIP3	Bcl_2_ interacting protein 3
CaMKIIδ	Ca^2+^/calmodulin-dependent protein kinase II delta
cMyBPc	cardiac myosin-binding protein c
Drp1	dynamin-related protein 1
FUNDC1	FUN14 Domain Containing 1
GSK2399872A	GSK’872
HR	heart rate
I/R	ischemia/reperfusion
IL-1β	interleukin-1 beta
JNK	c-Jun N-terminal kinase
LDH	lactate dehydrogenase
LVDP	left ventricular developed pressure
LVEDP	LV end-diastolic pressure
MLKL	mixed lineage kinase domain-like pseudokinase
MnSOD	manganese superoxide dismutase
mPTP	mitochondrial permeability transition pore
NLRP3	NOD-, LRR- and pyrin domain-containing protein 3
NOX2	NADPH oxidase 2
PGAM5	phosphoglycerate mutase family member 5
PLN	phospholamban
RIP1	receptor-interacting protein kinase 1
RIP3	receptor-interacting protein kinase 3
ROS	reactive oxygen species
RPP	rate pressure product
TNF	tumour necrosis factor
XO	xanthin oxidase
