# Supplementary material for: Inhibition of Cardiac RIP3 Mitigates Early Reperfusion Injury and Calcium-Induced Mitochondrial Swelling without Altering Necroptotic Signalling
Source: Int J Mol Sci. 2021 Jul 26;22(15):7983. doi: 10.3390/ijms22157983 (PMC8347133; doi:10.3390/ijms22157983)
Supplement: Supplementary file 1 [file ijms-22-07983-s001.zip › ijms-1288346-supplementary.pdf]

SUPPLEMENTARY MATERIAL

for

Inhibition of cardiac RIP3 mitigates early reperfusion injury and calcium-induced mitochondrial swelling without altering necroptotic signalling

Supplementary methods and Supplementary results

The detailed protocol of Western blot analysis is described in the main text of the manuscript. Here, we provide additional data and list the antibodies against proteins presented below as Supplementary Figures.

Antibodies used for immunoblotting: anti-caspase-8 (#4790 Cell Signaling Technology, USA), anti-caspase-3 (#9662 Cell Signaling Technology, USA), anti-Bcl2 (SAB4500003, Sigma-Aldrich, USA), anti-Bax (ab182734, Abcam, UK).

Procaspase-8 was increased due to I/R and RIP3 inhibition did not modify these levels. The same pattern of results was, however, not seen in case of its active form – caspase-8, which levels were comparable among the groups. The zymogen form of caspase-3, an executioner of apoptosis, was altered due to neither I/R nor RIP3 inhibition, while the signal for its cleaved form was totally absent. Data on the ratio of Bcl2/Bax, another reliable marker of the cells susceptibility to apoptosis, have also indicated that apoptosis was not active under our experimental settings (Supplementary Figure 1).

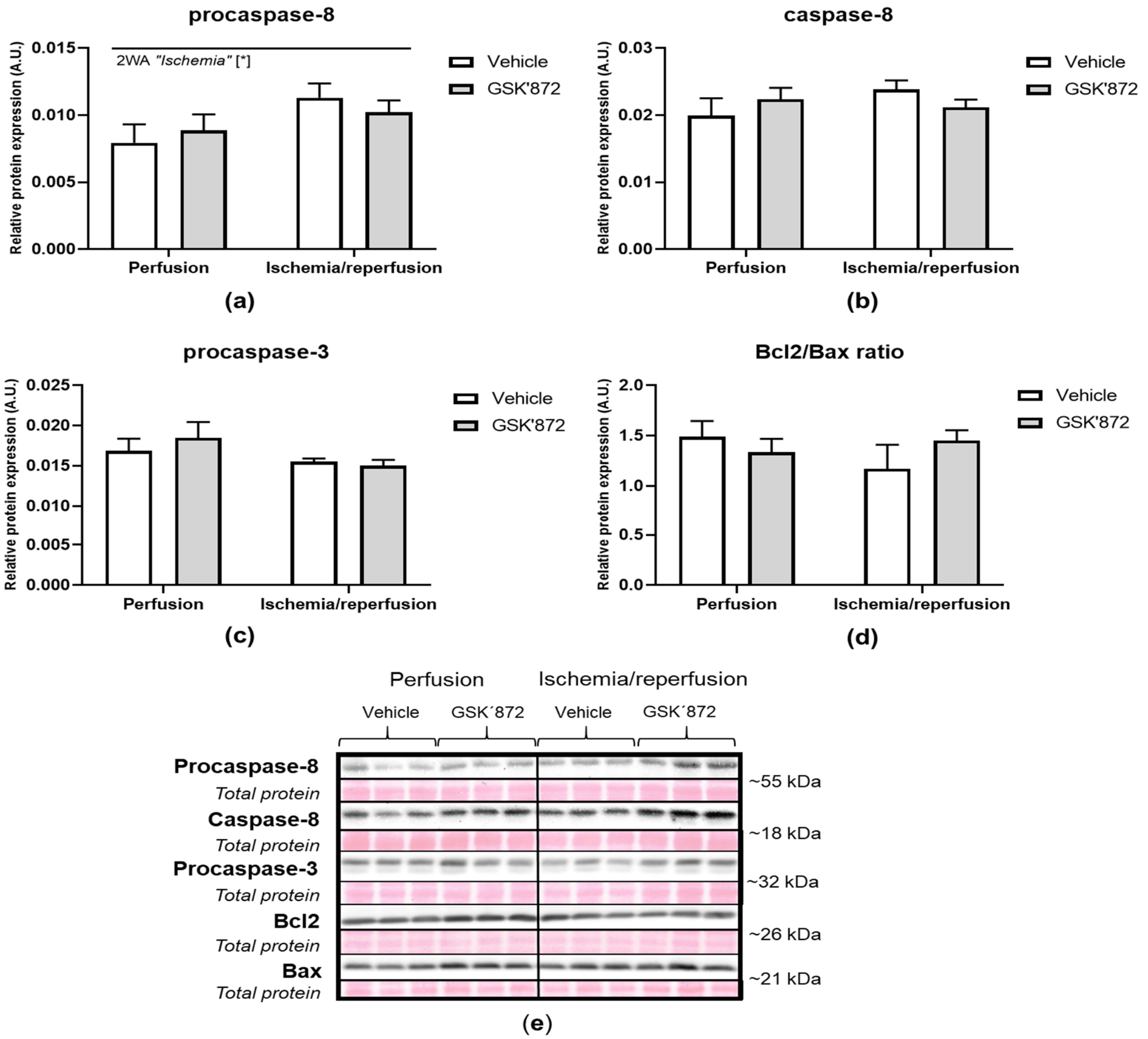

Figure S1. Caspase-8 and some apoptosis markers in rat hearts subjected to RIP3 inhibition.

Immunoblot quantification of procaspase-8 (**a**), caspase-8 (**b**), procaspase-3 (**c**), Bcl2/Bax ratio (**d**); Representative immunoblots and total protein staining (**e**). Data are presented as mean ± SEM; **P* < 0.05. 2WA—two-way ANOVA; factor "*Ischemia*"—presence of ischemia/reperfusion.
